# Hierarchical trie packet classification algorithm based on expectation-maximization clustering

**DOI:** 10.1371/journal.pone.0181049

**Published:** 2017-07-13

**Authors:** Xia-an Bi, Junxia Zhao

**Affiliations:** College of Mathematics and Computer Science, Hunan Normal University, Changsha, P.R. China; Universidad de Valladolid, SPAIN

## Abstract

With the development of computer network bandwidth, packet classification algorithms which are able to deal with large-scale rule sets are in urgent need. Among the existing algorithms, researches on packet classification algorithms based on hierarchical trie have become an important packet classification research branch because of their widely practical use. Although hierarchical trie is beneficial to save large storage space, it has several shortcomings such as the existence of backtracking and empty nodes. This paper proposes a new packet classification algorithm, Hierarchical Trie Algorithm Based on Expectation-Maximization Clustering (HTEMC). Firstly, this paper uses the formalization method to deal with the packet classification problem by means of mapping the rules and data packets into a two-dimensional space. Secondly, this paper uses expectation-maximization algorithm to cluster the rules based on their aggregate characteristics, and thereby diversified clusters are formed. Thirdly, this paper proposes a hierarchical trie based on the results of expectation-maximization clustering. Finally, this paper respectively conducts simulation experiments and real-environment experiments to compare the performances of our algorithm with other typical algorithms, and analyzes the results of the experiments. The hierarchical trie structure in our algorithm not only adopts trie path compression to eliminate backtracking, but also solves the problem of low efficiency of trie updates, which greatly improves the performance of the algorithm.

## Introduction

The core equipment of computer network is the router and firewall. Packet classification technology is the key technology of these core devices, which restricts the development of computer network bandwidth. Thus, packet classification technology has great significance on the next-generation Internet network equipment[1], and plays important roles in routing, quality of service, firewall, multimedia communications, accounting, traffic monitoring, and so on[2]. With the rapid development of high-speed network, packet classification technology has become one of the main factors that affect the improvement of network equipment[3]. Meanwhile, packet classification algorithms are required to deal with larger number of rule sets. Researches on efficient packet classification algorithms which support large-scale rule sets are of great significance[4].

The main entities of packet classification are packets and rules. Rules are defined as multiple fields of packet headers and actions. Fields are usually divided into five parts: source IP address prefixes, destination IP address prefixes, source port, destination port and protocols[5]. The role of packet classification is to distinguish the numerous data packets to different types based on rules and then deal with different types of packets with distinguishing actions, such as routing forward, packet filtering. Although packet classification technology exists in computer network equipment, it is an independent technology that needs to be studied. An effective packet classification technology needs to get rid of the shackles of network services and could be deployed in various devices.

Packet classification technology develops rapidly and diverse flows of packet classification algorithms have been proposed in the past decades. Nevertheless, most literature mainly focuses on the performance improvement of the packet classification algorithm, and neglects the theoretical analysis and the problems which occur in the implementation process[6–7]. In the background of high-speed network, packet classification algorithms are not required to have the only feature of intensive design tasks on time/space complexity, but also need to have good scalability and high flexibility to support large number of rules. Therefore, the performance evaluation of packet classification algorithms include several metrics, among which the processing speed and memory storage are the most fundamental and commonly-used. Incremental scalability and update performance of the algorithms have turned into another two important metrics, and become growing concerns in the existing literature[8–9].

Existing packet classification algorithms are divided into three flows: basic data structure algorithms[10–14], space mapping algorithms[15–19] and hardware-based algorithms[20–22]. Basic data structure algorithms and space mapping algorithms are featured with complex data structures, and easy to implement and deploy, but these two types of algorithms face the bottleneck of performance due to the complex data structures. Hardware-based algorithms usually use hardware such as TCAMs. This type of algorithms has high searching speed performance. However, these hardwares are expensive and do not support flexible scalability. Moreover, this type of algorithms are only suitable for small-scale rule sets because of the high energy consumption, which hinders their widespread use. Therefore, a new solution is required to achieve high scalability and update performance as well as high classification performance.

To fill out the research gap, this paper uses cluster analysis theory to construct Hierarchical Trie to solve the matching problems between packets and rules. Firstly, this paper uses the formalization method to deal with the packet classification problem by means of mapping the rules and data packets into a two-dimensional space. Secondly, this paper uses Expectation-Maximization algorithm to cluster the rules based on their aggregate characteristics, and thereby diversified clusters are formed. Thirdly, this paper proposes a hierarchical trie based on the results of expectation-maximization clustering. Finally, this paper respectively conducts simulation experiments and real-environment experiments to test the performances of the proposed algorithm, and analyzes the results of the experiments.

By combining expectation maximization algorithm and hierarchical tries, this paper makes the following contributions. In theory, we propose the formalization of the packet classification problem based on geometric space. This method uses mathematical models to map data packets and rules into the rectangular area in two-dimensional space. Then we use the theoretical analysis to prove the mathematical model established by this method, and conclude that the packets and rules still keep the original features and the mapping rectangular area still meets the packet matching process. In terms of algorithm, this paper design a novel hierarchical trie structure which not only adopts trie path compression to eliminate backtracking, but also solves the problem of update performance, and thereby the performance of the algorithm has been greatly improved. In practice, we deploy our algorithm in the network traffic monitoring system to test the performances of the algorithms and further improve our algorithm. The experimental results show that the proposed packet classification algorithm has high-speed packet classification performance, and low storage requirement. At the same time, it can be easily implemented and deployed.

The rest of this paper is organized as follows. Section 2 reviews the related works. In Section 3, the formalization of packet classification is presented in details. In section 4, a Hierarchical Trie Packet Classification Algorithm Based on Expectation-Maximization Clustering is proposed. Section 5 discusses the experimental evaluation, and Section 6 gives the conclusions.

## Related works

In this section, we provide a brief discussion on the packet classification algorithms. General packet classification algorithm are roughly divided into basic data structure algorithms, space mapping algorithms and hardware-based algorithms. The survey of the packet classification algorithms is shown in Table 1.

**Table 1:** The classification of the packet classification algorithms.

	Typical algorithms	Characteristics
**Basic data structure algorithms**	SPT,EGT-PC, RS, PDCBF[],AQT[]	Better scalability, Low performance
**Space mapping algorithms**	HiCuts [],HyperCuts[], RFC[], GroupCuts	High time performance, Large memory requirements
**Hardware-based algorithms**	TCAM-based algorithms, GPU-based algorithms, FPGA-based algorithms	High performance, costly, not easy to expand

### Basic data structure packet classification algorithms

Existing basic data structure packet classification algorithms are mainly divided into trie-based packet classification algorithms, tuple space-based packet classification algorithms and Bloom Filter- based packet classification algorithms. The representative algorithms are Set-Pruning Trie[10], Extended Grid of Trie with Path Compression[11], Rectangle Search[12], Parallel Distributed Combination Bloom Filter [13], Area-based Quad-Trie[14] and so on.

Basic data structure packet classification algorithms have better scalability, thereinto trie-based packet classification algorithms are widely used[23]. However, trie-based packet classification algorithms need to search for all possible matching rules by backtracking. When this type of algorithms are applied to IPv6, the performance significantly reduces. Therefore, we need to develop a data structure-based packet classification algorithm that supports fast-speed classification as well as large-size rule sets.

### Space mapping packet classification algorithms

Most space mapping packet classification algorithms fall into three main categories: geometric area-based packet classification algorithms, dimension decomposition-based packet classification algorithms and clustering-based packet classification algorithms. The representative algorithms are Hierarchical Intelligent Cuttings[15], HyperCuts[16], Recursive Flow Classification[17], GroupCuts[18], unsupervised co-clustering algorithm[19] and so on.

Space mapping packet classification algorithms take up less searching time but require large memory storage. This type of algorithms could not satisfy the requirements of high searching speed brought by Gigabit challenge[24]. However, due to their data structures’ requirement for storing a filter, the storage performances are significantly and negatively affected[25]. Clustering-based packet classification algorithms can solve the problem of backtracking, which exists in hierarchical trie packet classification algorithm. However, clustering-based packet classification algorithms also have several demerits such as low update performance of rules.

### Hardware-based packet classification algorithms

Existing hardware-based packet classification algorithms are mainly divided into Ternary content addressable memory (TCAM)-based algorithms[20], Graphic Processing Unit (GPU)-based algorithms[21], and Field-Programmable Gate Array (FPGA)-based algorithms[22].

TCAM-based packet classification algorithms, which are featured with parallel searches and matching result reports in a single cycle, are the preferred choice by the industry up till now. Because of the parallel operation, the high speed advantage always comes at a price like huge energy consumption[20]. FPGA-based packet classification algorithms are featured with reconfigurability. Although this kind of customized architecture provides high performance, it is not easy to implement and deploy [22]. In the field of high performance computing, general-purpose computing with GPU has become a new research trend. Such algorithms are featured with several types of memory storage and usage in various functions on the GPU[21]. However, how to effectively enhance the ability of parallelism is still a great challenge.

In conclusion, existing algorithms usually stand out in a certain aspect of performance, but little literature proposes the packet classification algorithms which are easy to implement and deploy and are featured with high speed performance, low storage requirements, flexible scalability and high update performance. Therefore, this paper propose a novel algorithm to solve the problem.

## Formalization of packet classification problem

### Rule formalization process

This paper formulates the packet classification problem as a mapping problem. It is assumed that the number of two-dimensional rules in a rule set R is n. Let SA, SA = {SA1, SA2 … SAn}, stand for the source IP prefixes, and DA, DA = {DA1, DA2 … DAn}, stand for the destination IP prefixes. For a rule Rm (m = 1, 2 … n) = {R1, R2, Rm … Rn}, the source IP prefix could be mapped to a range [$L_{R_{m}SA},H_{R_{m}SA}$] where $L_{R_{m}SA}$ and $H_{R_{m}SA}$ are respectively the lower and upper boundaries of the source IP prefix range, and the corresponding destination IP prefix could be mapped to a range [$L_{R_{m}DA},H_{R_{m}DA}$] where $L_{R_{m}DA}$ and $H_{R_{m}DA}$ are respectively the lower and upper boundaries of the destination IP prefix range. Then this rule have been mapped to a small rectangular area in the two-dimensional space.

Let us make the center point of this rectangle represent the rule, and thereby the rule Rm can be written as a point:

$$M\left((L_{R_{m}SA}+H_{R_{m}SA})/2,(L_{R_{m}DA}+H_{R_{m}DA})/2\right)$$

And we can obtain:

$$L_{R_{m}SA}=\sum_{i=1}^{w_{R}}\frac{k}{i\left(i+k\right)}VR_{i}$$ (1)

$$H_{R_{m}SA}=\sum_{i=1}^{w_{R}}\frac{k}{i\left(i+k\right)}VR_{i}+\frac{1}{w_{R}+1}+\frac{1}{w_{R}+2}+\cdots +\frac{1}{w_{R}+k}$$ (2)

$$L_{R_{m}DA}=\sum_{i=1}^{w_{R}}\frac{k}{i\left(i+k\right)}VR_{i}$$ (3)

$$H_{R_{m}DA}=\sum_{i=1}^{w_{R}}\frac{k}{i\left(i+k\right)}VR_{i}+\frac{1}{w_{R}+1}+\frac{1}{w_{R}+2}+\cdots +\frac{1}{w_{R}+k}$$ (4)

where *w*_*R*_ is the prefix length of *R*_*m*_, *VR*_*i*_ is the value of i-th bit in the prefix of *R*_*m*_ (*VR*_*i*_ is either 0 or 1), *k* is any positive integer.

### Packet formalization process

It is assumed that SAp stands for the packet source IP address, and DAp stands for the destination IP address. The source IP address could be mapped to a range [*L*_*PSA*_, *H*_*PSA*_] where *L*_*PSA*_ and *H*_*PSA*_ are respectively the lower and upper boundaries of the source IP address range, and the corresponding destination IP address could be mapped to a range [*L*_*PDA*_, *H*_*PDA*_] where *L*_*PDA*_ and *H*_*PDA*_ are respectively the lower and upper boundaries of the destination IP address range. Then this packet have been mapped to a smaller rectangular area in the two-dimensional space compared with the rule Rm.

Let us make the center point of this rectangle represent the packet, and thereby the packet P can be written as a point:

$$M\left((L_{PSA}+H_{PSA})/2,(L_{PDA}+H_{PDA})/2\right)$$

And we can obtain:

$$L_{PSA}=\sum_{i=1}^{w_{P}}\frac{k}{i\left(i+k\right)}VP_{i}$$ (5)

$$H_{PSA}=\sum_{i=1}^{w_{P}}\frac{k}{i\left(i+k\right)}VP_{i}+\frac{1}{w_{P}+1}+\frac{1}{w_{P}+2}+\cdots +\frac{1}{w_{P}+k}$$ (6)

$$L_{PDA}=\sum_{i=1}^{w_{P}}\frac{k}{i\left(i+k\right)}VP_{i}$$ (7)

$$H_{PDA}=\sum_{i=1}^{w_{P}}\frac{k}{i\left(i+k\right)}VP_{i}+\frac{1}{w_{P}+1}+\frac{1}{w_{P}+2}+\cdots +\frac{1}{w_{P}+k}$$ (8)

where *w*_*P*_ is the address length of packet P (i.e., *w*_*P*_ = 32 in IPv4), *VP*_*i*_ is the value of i-th bit in address of packet P (*VP*_*i*_ is either 0 or 1).

### Packet matching formalization process

Packet matching process is a matching process between packets and the rules in the rule set. Specifically, the aim of packet matching process is to find the matching rules in accordance with one or more packet header fields, and then perform the appropriate actions. In this paper, we use the prefix matching which is the most widely-used and important among all the matching types.

**Lemma 1**. *If a packet P matches with the rule R*_*m*_, *then* $\left[L_{PSA},H_{PSA}]\subset [L_{R_{m}SA},H_{R_{m}SA}\right]$ *and* $\left[L_{PDA},H_{PDA}]\subset [L_{R_{m}DA},H_{R_{m}DA}\right]$.

*Proof* If the packet P matches with the rule *R*_*m*_, we can infer that the values of the first j-bit of rule *R*_*m*_ are the same as the values of the first j-bit of packet P, that is, *VP*_1_ = *VR*_1_, *VP*_2_ = *VR*_2_, … …, *VP*_*j*_ = *VR*_*j*_. Moreover, we can infer that the bit length j of the same values equals to the prefix length *w*_*R*_ of *R*_*m*_, that is, *j* = *w*_*R*_.

Because

$$\begin{array}{c} L_{PSA}=\sum_{i=1}^{w_{P}}\frac{k}{i\left(i+k\right)}VP_{i}\\ =\sum_{i=1}^{j}\frac{k}{i\left(i+k\right)}VP_{i}+\sum_{i=j+1}^{w_{P}}\frac{k}{i\left(i+k\right)}VP_{i}\\ \geq \sum_{i=1}^{j}\frac{k}{i\left(i+k\right)}VR_{i}=L_{R_{m}SA} \end{array}$$ (9)

And

$$\begin{array}{l} H_{PSA}=\sum_{i=1}^{w_{P}}\frac{k}{i\left(i+k\right)}VP_{i}+\frac{1}{w_{P}+1}+\frac{1}{w_{P}+2}+\cdots +\frac{1}{w_{P}+k}\\ =\sum_{i=1}^{j}\frac{k}{i\left(i+k\right)}VP_{i}+\sum_{i=j+1}^{w_{P}}\frac{k}{i\left(i+k\right)}VP_{i}+\frac{1}{w_{P}+1}+\frac{1}{w_{P}+2}+\cdots +\frac{1}{w_{P}+k} \end{array}$$ (10)

If the values of packet P equals to 1 from the j+1-th bit to the last bits, the original formula becomes

$$\begin{array}{l} H_{PSA}\leq \sum_{i=1}^{j}\frac{k}{i\left(i+k\right)}VP_{i}+\left(\frac{1}{j+1}+\frac{1}{j+2}+\cdots +\frac{1}{j+k}\right)-\left(\frac{1}{w_{P}+1}+\frac{1}{w_{P}+2}+\cdots +\frac{1}{w_{P}+k}\right)+\frac{1}{w_{P}+1}+\frac{1}{w_{P}+2}+\\ \cdots +\frac{1}{w_{P}+k}=\sum_{i=1}^{j}\frac{k}{i\left(i+k\right)}VP_{i}+\frac{1}{j+1}+\frac{1}{j+2}+\cdots +\frac{1}{j+k} \end{array}$$ (11)

As *j* = *w*_*R*_, we can get $H_{PSA}\leq H_{R_{m}SA}$.

Similarly, we can get $L_{PSA}\geq L_{R_{m}SA}$. Then the conclusion $\left[L_{PSA},H_{PSA}]\subset [L_{R_{m}SA},H_{R_{m}SA}\right]$ can be obtained. The conclusion $\left[L_{PDA},H_{PDA}]\subset [L_{R_{m}DA},H_{R_{m}DA}\right]$ could be proved in the same way.

## Hierarchical trie algorithm based on expectation-maximization clustering

This section proposed a hierarchical trie algorithm for packet classification based on expectation-maximization clustering. The algorithm has two stages, one is the preprocessing stage of rules and packets, one is the packet matching stage. In the first stage, we firstly adopt the formalization method of packet classification problem to map the rules and packets into rectangular area in the two-dimensional space. Then we use expectation-maximization algorithm to cluster the formalized rules and thus a plurality of clusters could be formed. In the second stage, we construct a hierarchical trie based on the existing clusters and complete the packet matching process. The hierarchical trie structure in this algorithm adopts the path compression to eliminate backtracking and overcomes the difficulty of trie update, which greatly improves the performance of the proposed algorithm.

### The main idea of HTEMC

Expectation Maximization (EM) algorithm is a framework which approximates the maximum likelihood estimate or the maximum a posteriori estimation of statistical model parameters. The EM algorithm is featured with many iterations, and it can make the algorithm to achieve the optimal state quickly. Each iteration is composed of two steps, expectation step and maximization step. In the expectation step, the subject is assigned to the corresponding cluster according to the parameters of the clusters. In the maximization step, new clusters or parameters could be found by minimizing the quadratic sum of fuzzy clustering error or the expectation likelihood of clusters based on the probability model [18]. The clusters, which are formed by using the expectation maximization method, are featured with high cohesion and low coupling. After employing this method, if a rule which matches the packet is found in a sub-trie, there is no need to search for other sub-tries Thus, the application of this method in the packet classification algorithm can largely save the packet’s searching time, improve time performance and also save memory space.

The specific steps of expectation maximization algorithm are as follows:

(1) Initialization. The number of convergence clusters does not vary with the changing numbers of initial clusters. The initialized methods have been discussed in the literature[26–29]. Based on the existing methods, we select the method in which the number of clusters is decided by the size of rule set. If the number of rules in the rule set is less than 1000, the initialized number of clusters is generally set as 100. If the number of rules is greater than 1000, but less than 10000, the initialized number of cluster is generally set as 1000.
(2) E- step. We first calculate each rule’s degree of membership with each cluster. Then we assign each rule R to the corresponding cluster *C*_*i*_ where i represents the i-th cluster on the basis of the membership weight $W_{R,C_{i}}$ between rule R and cluster *C*_*i*_. Let *dist*(*R*, *C*_*i*_) denote the Euclidean distance of rule R and cluster *C*_*i*_. If rule R is close to cluster *C*_*i*_, then *dist*(*R*, *C*_*i*_) is small, and the degree of the membership between R on *C*_*i*_ is high. We normalized all the degrees of membership, and make the sum of the degree membership of each rule equal to 1. It is assumed that the number of clusters is n, then we can get
  $${\text{W}}_{\text{R},{\text{C}}_{\text{i}}}=\frac{1/\text{dist}{\left(\text{R},{\text{C}}_{\text{i}}\right)}^{2}}{\left(1/\text{dist}{\left(\text{R},{\text{C}}_{1}\right)}^{2}+1/\text{dist}{\left(\text{R},{\text{C}}_{2}\right)}^{2}+\cdots +1/\text{dist}{\left(\text{R},{\text{C}}_{\text{i}}\right)}^{2}+\cdots +1/\text{dist}{\left(\text{R},{\text{C}}_{\text{n}}\right)}^{2}\right)}$$ (12)

Table 2 shows the two-dimensional rule table sample, and Table 3 shows the formalized packet classification.

**Table 2:** A two-dimensional rule table sample.

Rule	Source IP Prefix	Destination IP Prefix
**R0**	0*	1*
**R1**	10*	0*
**R2**	11*	1*
**R3**	00*	11*
**R4**	1*	1*
**R5**	010*	011*
**R6**	01*	010*

**Table 3:** The rule set after formalization.

Rule	Source IP Prefix	Destination IP Prefix	${\text{L}}_{{\text{R}}_{\text{m}}\text{SA}}$	${\text{H}}_{{\text{R}}_{\text{m}}\text{SA}}$	${\text{L}}_{{\text{R}}_{\text{m}}\text{DA}}$	${\text{H}}_{{\text{R}}_{\text{m}}\text{DA}}$	M	12 M
**R0**	0*	1*	0	1/2	1/2	1	(1/4,3/4)	(3,9)
**R1**	10*	0*	1/2	5/6	0	1/2	(2/3,1/4)	(8,3)
**R2**	11*	1*	2/3	1	1/2	1	(5/6,3/4)	(10,9)
**R3**	00*	11*	0	1/3	2/3	1	(1/6,5/6)	(2,10)
**R4**	1*	1*	1/2	1	1/2	1	(3/4,3/4)	(9,9)
**R5**	010*	011*	1/6	5/12	3/12	9/12	(7/24,1/2)	(3.5,6)
**R6**	01*	010*	1/6	1/2	1/6	5/12	(1/3,7/24)	(4,3.5)

We shall assume n = 3 for the rule in Table 2, that is, there are three clusters in the initial stage. Let R4, R5, R6 respectively denote the initial cluster centers of the three clusters. Then we can get

$${\text{W}}_{\text{R},{\text{C}}_{1}}=\frac{1/\text{dist}{\left(\text{R},{\text{C}}_{1}\right)}^{2}}{\left(1/\text{dist}{\left(\text{R},{\text{C}}_{1}\right)}^{2}+1/\text{dist}{\left(\text{R},{\text{C}}_{2}\right)}^{2}+1/\text{dist}{\left(\text{R},{\text{C}}_{3}\right)}^{2}\right)}$$ (13)

$${\text{W}}_{\text{R},{\text{C}}_{2}}=\frac{1/\text{dist}{\left(\text{R},{\text{C}}_{2}\right)}^{2}}{\left(1/\text{dist}{\left(\text{R},{\text{C}}_{1}\right)}^{2}+1/\text{dist}{\left(\text{R},{\text{C}}_{2}\right)}^{2}+1/\text{dist}{\left(\text{R},{\text{C}}_{3}\right)}^{2}\right)}$$ (14)

$${\text{W}}_{\text{R},{\text{C}}_{3}}=\frac{1/\text{dist}{\left(\text{R},{\text{C}}_{3}\right)}^{2}}{\left(1/\text{dist}{\left(\text{R},{\text{C}}_{1}\right)}^{2}+1/\text{dist}{\left(\text{R},{\text{C}}_{2}\right)}^{2}+1/\text{dist}{\left(\text{R},{\text{C}}_{3}\right)}^{2}\right)}$$ (15)

The first iteration is as follows:

$$\begin{array}{ccc} \text{dist}{\left({\text{R}}_{0},{\text{C}}_{1}\right)}^{2}=36 & \text{dist}{\left({\text{R}}_{0},{\text{C}}_{2}\right)}^{2}=9.25 & \text{dist}{\left({\text{R}}_{0},{\text{C}}_{3}\right)}^{2}=27.75\\ {\text{W}}_{{\text{R}}_{0},{\text{C}}_{1}}=0.16 & {\text{W}}_{{\text{R}}_{0},{\text{C}}_{2}}=0.63 & {\text{W}}_{{\text{R}}_{0},{\text{C}}_{3}}=0.21\\ \text{dist}{\left({\text{R}}_{1},{\text{C}}_{1}\right)}^{2}=37 & \text{dist}{\left({\text{R}}_{1},{\text{C}}_{2}\right)}^{2}=29.25 & \text{dist}{\left({\text{R}}_{1},{\text{C}}_{3}\right)}^{2}=16.25\\ {\text{W}}_{{\text{R}}_{1},{\text{C}}_{1}}=0.22 & {\text{W}}_{{\text{R}}_{1},{\text{C}}_{2}}=0.28 & {\text{W}}_{{\text{R}}_{1},{\text{C}}_{3}}=0.50\\ \text{dist}{\left({\text{R}}_{2},{\text{C}}_{1}\right)}^{2}=1 & \text{dist}{\left({\text{R}}_{2},{\text{C}}_{2}\right)}^{2}=51.25 & \text{dist}{\left({\text{R}}_{2},{\text{C}}_{3}\right)}^{2}=66.25\\ {\text{W}}_{{\text{R}}_{2},{\text{C}}_{1}}=0.97 & {\text{W}}_{{\text{R}}_{2},{\text{C}}_{2}}=0.02 & {\text{W}}_{{\text{R}}_{2},{\text{C}}_{3}}=0.01\\ \text{dist}{\left({\text{R}}_{3},{\text{C}}_{1}\right)}^{2}=50 & \text{dist}{\left({\text{R}}_{3},{\text{C}}_{2}\right)}^{2}=18.25 & \text{dist}{\left({\text{R}}_{3},{\text{C}}_{3}\right)}^{2}=46.25\\ {\text{W}}_{{\text{R}}_{3},{\text{C}}_{1}}=0.21 & {\text{W}}_{{\text{R}}_{3},{\text{C}}_{2}}=0.57 & {\text{W}}_{{\text{R}}_{3},{\text{C}}_{3}}=0.22\\ \text{dist}{\left({\text{R}}_{4},{\text{C}}_{1}\right)}^{2}=0 & \text{dist}{\left({\text{R}}_{4},{\text{C}}_{2}\right)}^{2}=39.25 & \text{dist}{\left({\text{R}}_{4},{\text{C}}_{3}\right)}^{2}=55.25\\ {\text{W}}_{{\text{R}}_{4},{\text{C}}_{1}}=0 & {\text{W}}_{{\text{R}}_{4},{\text{C}}_{2}}=0.58 & {\text{W}}_{{\text{R}}_{4},{\text{C}}_{3}}=0.42\\ \text{dist}{\left({\text{R}}_{5},{\text{C}}_{1}\right)}^{2}=39.25 & \text{dist}{\left({\text{R}}_{5},{\text{C}}_{2}\right)}^{2}=0 & \text{dist}{\left({\text{R}}_{5},{\text{C}}_{3}\right)}^{2}=6.5\\ {\text{W}}_{{\text{R}}_{5},{\text{C}}_{1}}=0.14 & {\text{W}}_{{\text{R}}_{5},{\text{C}}_{2}}=0 & {\text{W}}_{{\text{R}}_{5},{\text{C}}_{3}}=0.86\\ \text{dist}{\left({\text{R}}_{6},{\text{C}}_{1}\right)}^{2}=55.25 & \text{dist}{\left({\text{R}}_{6},{\text{C}}_{2}\right)}^{2}=6.5 & \text{dist}{\left({\text{R}}_{6},{\text{C}}_{3}\right)}^{2}=0\\ {\text{W}}_{{\text{R}}_{6},{\text{C}}_{1}}=0.11 & {\text{W}}_{{\text{R}}_{6},{\text{C}}_{2}}=0.89 & {\text{W}}_{{\text{R}}_{6},{\text{C}}_{3}}=0 \end{array}$$

Then we can get the membership weighted matrix

$${\text{M}}^{\text{T}}=\left(\begin{array}{lllllll} 0.16 & 0.22 & 0.97 & 0.21 & 0 & 0.14 & 0.11\\ 0.63 & 0.28 & 0.02 & 0.57 & 0.58 & 0 & 0.89\\ 0.21 & 0.50 & 0.01 & 0.22 & 0.42 & 0.86 & 0 \end{array}\right)$$

(3) M- step. We recalculate the cluster centers based on the membership weighted matrix, and the new cluster center can be rewritten as
  $${\text{C}}_{\text{i}}=\frac{\sum_{\text{R}}{\text{W}}^{2}{}_{\text{R},{\text{C}}_{\text{i}}}{\text{M}}_{\text{R}}}{\sum_{\text{R}}{\text{W}}^{2}{}_{\text{R},{\text{C}}_{\text{i}}}}$$ (16)

Then we repeat this iteration, and each iteration contains an E-step and an M-step. Table 4 shows the results of the first four iterations. The final three clusters formed after the iterations are C1 {R2, R4}, C2 {R0, R3}, C3 {R1, R5, R6}. When the cluster center converges or changes to sufficiently small, the algorithm stops.

**Table 4:** The results of the four iterations.

Iteration	E-step	M-step
**1**	$\left(\begin{array}{lllllll} 0.16 & 0.22 & 0.97 & 0.21 & 0 & 0.14 & 0.11\\ 0.63 & 0.28 & 0.02 & 0.57 & 0.58 & 0 & 0.89\\ 0.21 & 0.50 & 0.01 & 0.22 & 0.42 & 0.86 & 0 \end{array}\right)$	*C*_1_: (9.24, 8.66) *C*_2_: (4.49, 6.67) *C*_3_: (5.09, 6.08)
**2**	$\left(\begin{array}{ccccccc} 0.1 & 0.24 & 0.96 & 0.15 & 0.98 & 0.02 & 0.08\\ 0.5 & 0.31 & 0.02 & 0.5 & 0.01 & 0.62 & 0.40\\ 0.4 & 0.45 & 0.02 & 0.35 & 0.01 & 0.36 & 0.52 \end{array}\right)$	*C*_1_: (9.31, 8.82) *C*_2_: (3.51, 6.93) *C*_3_: (4.39, 5.65)
**3**	$\left(\begin{array}{lllllll} 0.08 & 0.27 & 0.97 & 0.13 & 0.99 & 0.01 & 0.06\\ 0.68 & 0.26 & 0.01 & 0.59 & 0.004 & 0.51 & 0.27\\ 0.24 & 0.47 & 0.02 & 0.28 & 0.006 & 0.48 & 0.67 \end{array}\right)$	*C*_1_: (9.34, 8.78) *C*_2_: (3.16, 7.98) *C*_3_: (4.54, 4.75)
**4**	$\left(\begin{array}{lllllll} 0.024 & 0.25 & 0.98 & 0.08 & 0.9911 & 0.04 & 0.03\\ 0.928 & 0.18 & 0.01 & 0.8 & 0.0046 & 0.38 & 0.08\\ 0.048 & 0.57 & 0.01 & 0.12 & 0.0043 & 0.58 & 0.89 \end{array}\right)$	*C*_1_: (9.73, 9.1) *C*_2_: (2.77, 9.01) *C*_3_: (4.75, 4.03)

### The building process of HTEMC

Based on the final three clusters C1 {R2, R4}, C2 {R0, R3}, C3 {R1, R5, R6}, we build three sub-tries. In each cluster, the prefixes which have prefix relationship with others are sorted by the prefix length in ascending order. Among the prefixes with prefix relationship, the prefix with smallest length is set as the root node, and the rest prefixes are inserted into the left sub-trie by the ascending prefix length. The prefixes without prefix relationship are inserted into the right sub-trie. We take the cluster C3 as an illustration to specify the process of building the sub-trie.

**Fig 1:**
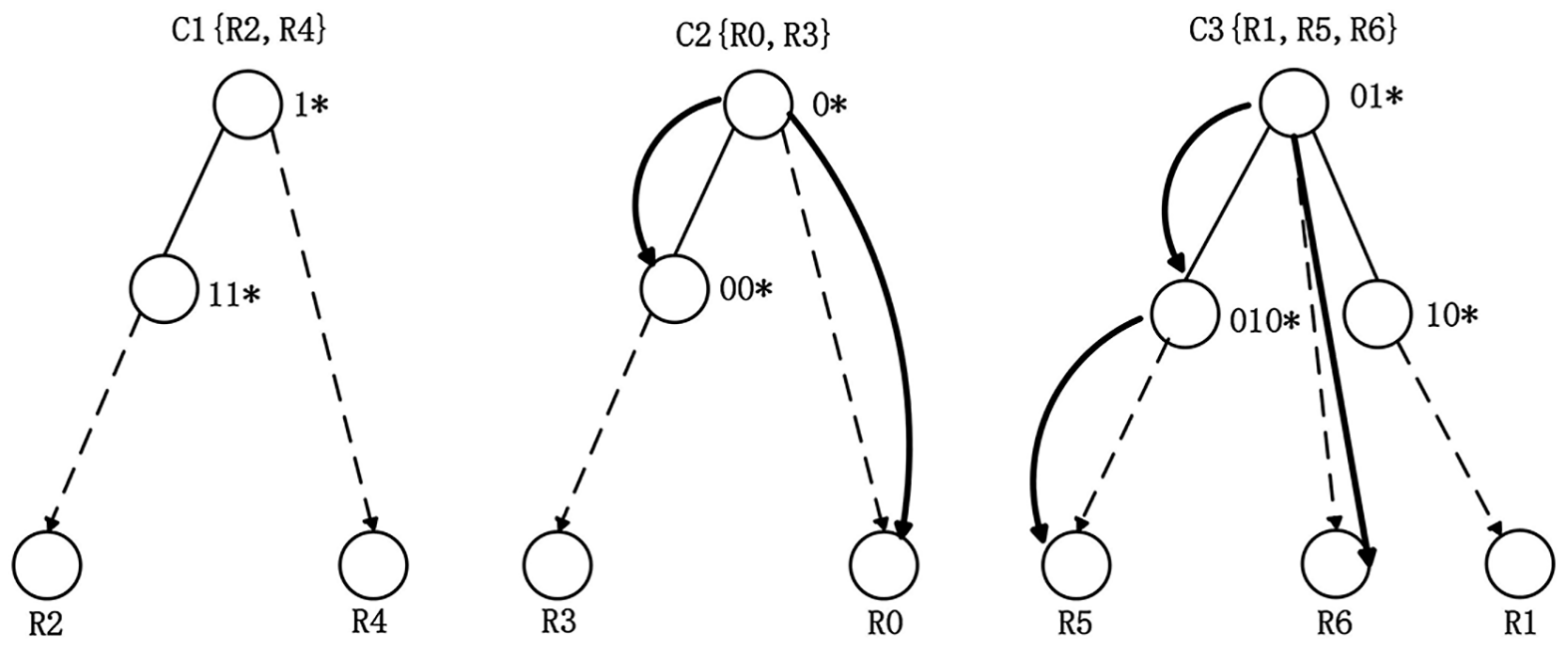
The structure and search process of HTEMC algorithm.

**Fig 2:**
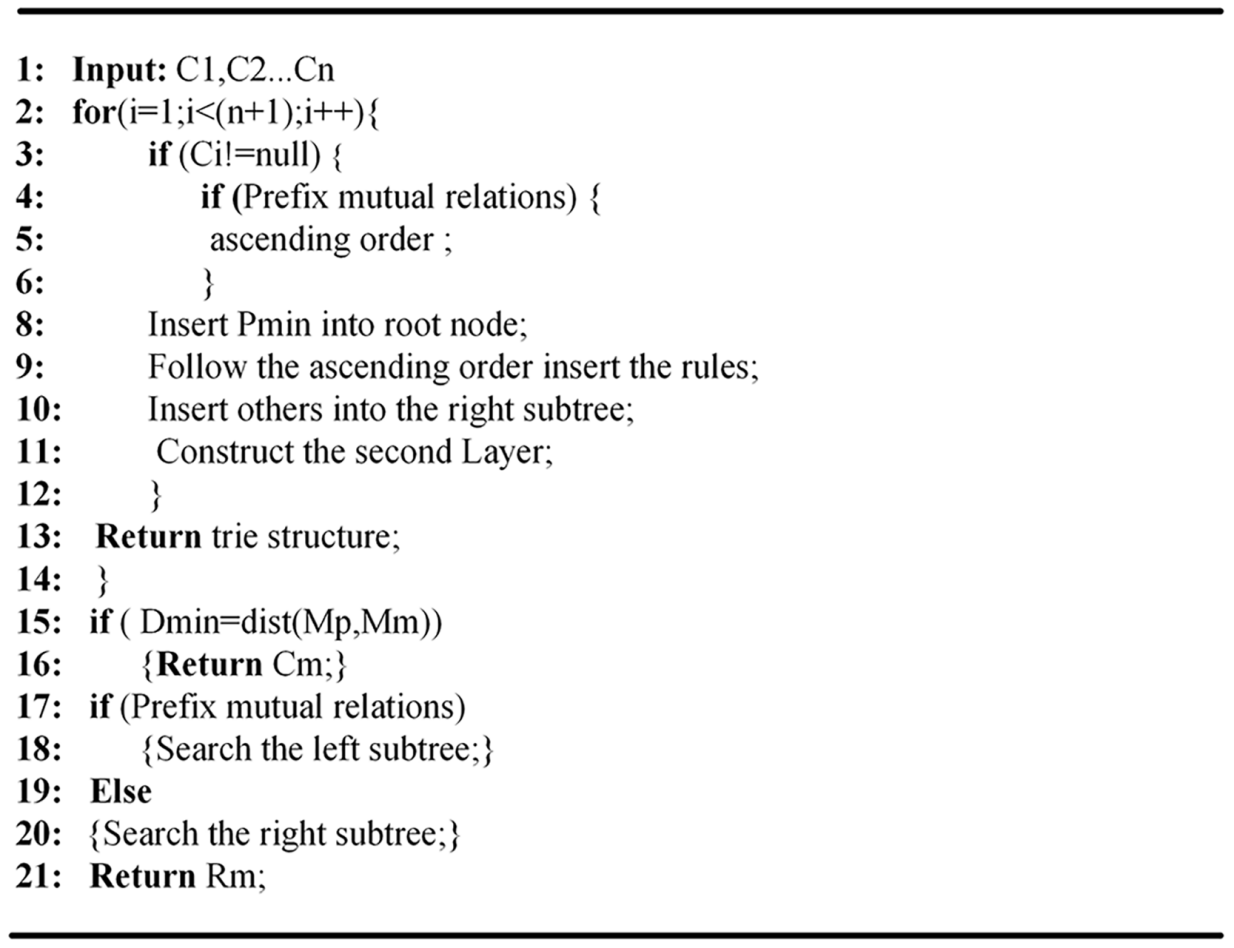
Pseudo-code of HTEMC algorithm.

In the cluster C3, 01 * and 010 * have the prefix relationship with each other. The prefix length of 01 *is 2, and the prefix length of 010*is 3. Thus we set 01 * as the root node, and insert 010 * into the left subtrie. Then we insert the prefix without the non-prefix relationship 10 * into the right sub-trie. After building the first layer of the trie, we adopt the direct insertion method to construct the second layer of the trie according to the destination IP address. Constructions of other sub-tries follow the same approach. It should be noted that the root node of each sub-trie needs to have a variable to store the point coordinates of the cluster center for the transformation of the trie structure when the rule set updates. The structure and searching process of HTEMC algorithm are shown in Fig 1, and the pseudo-code of HTEMC algorithm is shown in Fig 2.

### The searching process of HTEMC

The EM clustering method finally gathers the rules with prefix membership in the same cluster, and the rules without prefix membership in different clusters. Therefore, if a rule which matches the packets is found in a sub-trie, there is no need to search for other sub-tries, which largely saves the searching time.

**Fig 3:**
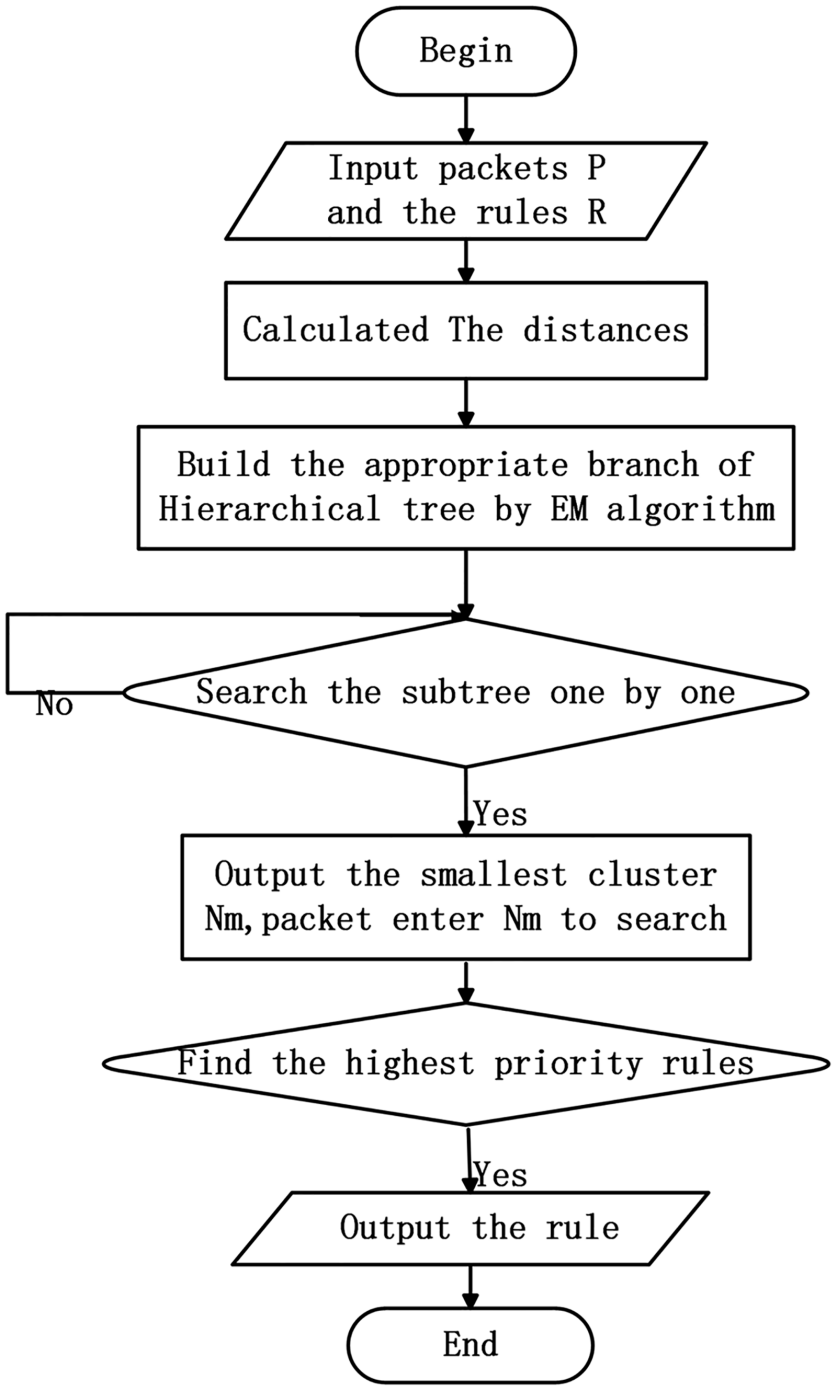
The flowchart of HTEMC algorithm.

The following example illustrates the searching process of Hierarchical Trie based on Expectation-Maximization Clustering. For the source IP prefix 010 * in packet (0101011,0110101), our algorithm initially searches the root node of the first sub-trie, and finds that 1 * does not match 010 *. Then it directly goes to the right of the first sub-trie to search and finds that the right of the first sub-trie is empty. Instantly, our algorithm goes the second sub-trie to search. It firstly finds that the root node 0 * of the second sub-trie matches 010 *. It enters the corresponding destination IP and finds that the rule R0 does not match the packet. Then it goes to the left of the second sub-trie to search and finds that 00 * does not match 010 *, and 00 * is the first layer of the leaf node. Afterwards, it directly goes to the root node of the third sub-trie. It finds that 01 *match 010 *, and then enters the corresponding destination IP but finds no matching. It then goes to the left of the third sub-trie and finds that 010 * matches 010 *. Finally, it enters the corresponding destination IP and finds that the rule R5 matches the packet and this node is a leaf node. The searching process is finished. The black arrows in Fig 1 shows the packet searching process in the trie and finally the longest matching rule R5 is obtained. The flowchart of HTEMC algorithm is shown in Fig 3.

### The updating process of HTEMC

Efficient packet classification algorithms are widely used in routers, firewalls and network monitoring systems and other network devices. Along with the development of the network, routers, firewalls and network monitoring system are facing new requirements, and thereby high update performance of rule sets becomes a main challenge for efficient packet classification algorithms.

Although existing hierarchical trie have high performance in search and storage, most of them can not overcome the update difficulty of rule sets. The update process of these algorithms needs to reconstruct the searching data structures when the rule set changes, which consumes much time and space. By contrast, our EMRCHT algorithm simply needs to transform searching data structures when the rule set changes instead of reconstructing the searching data structures. The updating idea of our EMRCHT algorithm is that when the rule set needs to add new rules, the algorithm only needs to formalize the new rule and calculate its distances to all the cluster center points. The new rule will be added to the sub-trie in the nearest cluster. In the process of transforming the searching data structure, the new rule is inserted into the left sub-trie if its source IP prefix has prefix membership with the root node of the trie, otherwise it is inserted into the right sub-trie.

The symbols and their definitions mentioned above are summarized in Table 5.

**Table 5:** Symbols and their definitions.

Symbol	Definition
n	the number of two-dimensional rules in a rule set
Rm (m = 1, 2 … n) = {R1, R2, Rm… Rn}	a rule set
SA = {SA1, SA2 … SAn}	the source IP prefixes
DA = {DA1, DA2 … DAn}	the destination IP prefixes
${\text{L}}_{{\text{R}}_{\text{m}}\text{SA}}$	the lower boundary of the source IP prefix range
${\text{H}}_{{\text{R}}_{\text{m}}\text{SA}}$	the upper boundary of the source IP prefix range
${\text{L}}_{{\text{R}}_{\text{m}}\text{DA}}$	the lower boundary of the destination IP prefix range
${\text{H}}_{{\text{R}}_{\text{m}}\text{DA}}$	the upper boundary of the destination IP prefix range
w_R_	the prefix length of Rm
VR_i_	the ith bit of prefix Rm (VR_i_ is either 0 or 1)
K	any positive integer
P	A packet
SAp	the packet source IP address
DAp	the destination IP address
L_PSA_	the lower boundary of the source IP address range
H_PSA_	the upper boundary of the source IP address range
L_PDA_	the lower boundary of the destination IP address range
H_PDA_	the upper boundary of the destination IP address range
w_P_	the address length of packet P
VP_i_	the i-th bit of address of packet P(VP_i_ is either 0 or 1)

## Performance evaluation in simulation environment

In this section, we compare our proposed algorithm with PTIAL algorithm by running a series of experiments to compare the performances of these two algorithms. The experiments are conducted by simulation on the ClassBench[30] platform. ClassBench provides classification tables which are similar to real classifiers in the Internet routers, and is able to input traces in accordance with each classification table. Specifically, we have performed simulations by using three different types of classification tables generated by ClassBench, access control lists (ACL), firewalls (FW), and IP chains (IPC). In ClassBench platform, it is the module ‘Filter Set Generator’ that produces synthetic rule sets. The synthetic rule sets can accurately model the characteristics of real rule sets. Though the size of the real rule sets varies, high-level control is provided by ClassBench and ClassBench can generate packet classification rule sets with different characteristics by setting parameters. We use it to generate traces which can simulate the traces running on routers and firewalls. Moreover, we do not set the distributions of protocol, port number and address in order to keep the authenticity of our experiments.

We mentioned four performance metrics of packet classification in the Introduction. In this section, we select three major metrics to evaluate algorithms’ performances in terms of searching speed, memory storage and updating performance. The searching speed, that is, the number of nodes which packets access, is an important metric to measure the time performance of an algorithm. The memory space that an algorithm costs is an essential metric to measure the space performance of the algorithm. We also use the time that a update costs to measure the update performance.

The detailed experiment scheme is as follows. We used ClassBench platform to generate two types of classifiers. One type is classifiers with big rule sets, and the sizes range from 500 to 5000 with an increase by 500. The other type is classifiers with small rule sets, and the sizes range from 100 to 1000 with an increase by 100. We respectively use these two types of classifiers to conduct the experiments and get the experimental results. It is noted that the trace generation rate is 1Gbits/sec, and background traffic is an exponential model in the experiment configuration.

### Searching speed performance comparison

**Fig 4:**
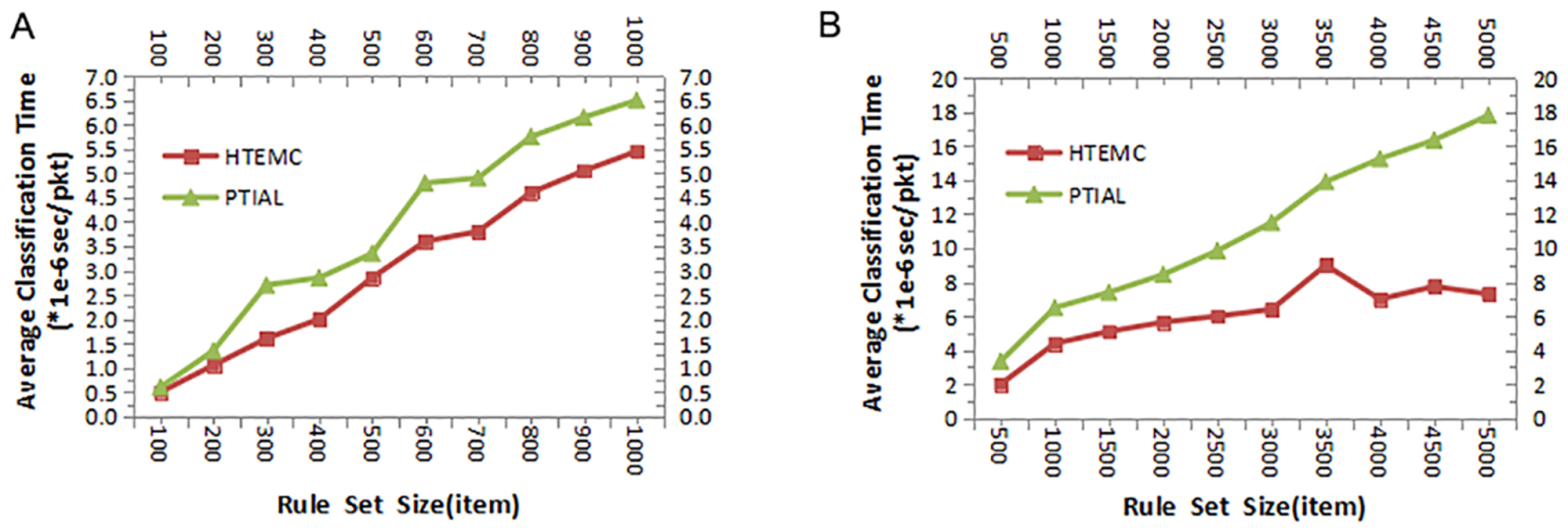
The comparisons of searching speed performances under different sizes of rule sets. (A)The average classification time of HTEMC algorithm and PTIAL algorithm were compared when the number of rules increases from 100 to 1000. (B)The average classification time of HTEMC algorithm and PTIAL algorithm were compared when the number of rules increases from 500 to 5000.

The comparisons of searching speed performances are presented in Fig 4. As seen from Fig 4A, when the size of rule set is small, the difference of searching speed performances between the two algorithms is not great, and our HTEMC algorithm has better time performance. When the number of rules increases to 1000, the average searching time of HTEMC algorithm reduces by 20% in comparison with PTIAL algorithm.

As shown in Fig 4B, when the size of rule set becomes large, the time performance advantage of HTEMC algorithm is much more obvious than PTIAL algorithm. When the number of rules increases to 5000, the average searching time of HTEMC algorithm reduces by 52% in comparison with PTIAL algorithm. Therefore, the time performance advantage in the searching speed of our HTEMC algorithm gradually stands out as the size of rule set increases.

### Memory performance comparison

**Fig 5:**
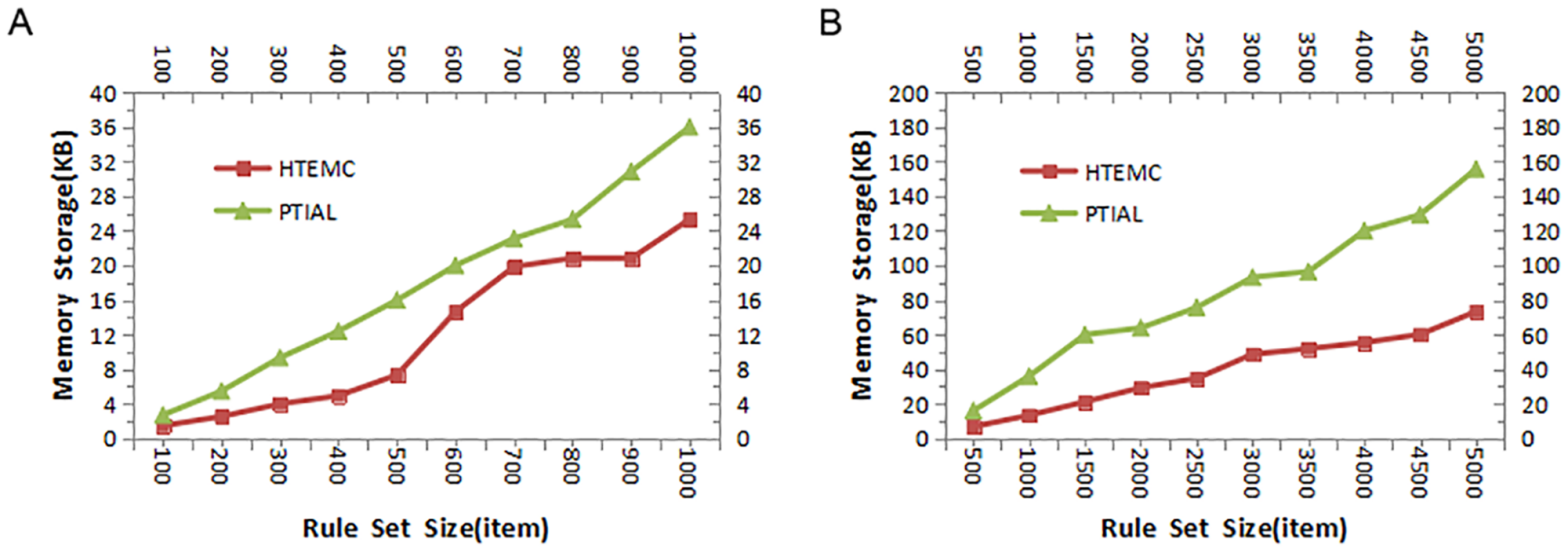
The comparisons of memory performances under different sizes of rule sets. (A)The memory storages of HTEMC algorithm and PTIAL algorithm were compared when the number of rules increases from 100 to 1000. (B)The memory storages of HTEMC algorithm and PTIAL algorithm were compared when the number of rules increases from 500 to 5000.

The comparisons of memory performances are presented in Fig 5. Fig 5A shows the comparison of the algorithms when the sizes of rule sets are small. In this scenario, as the size of the rule set is small which would not take up much memory, the space performance advantage of our HTEMC algorithm is not significant. When the number of rules increases to 1000, the average memory usage of HTEMC algorithm reduces by 25% in comparison with PTIAL algorithm.

Fig 5B shows the comparison of the algorithms when the sizes of rule sets are big. In the scenario, the space performance advantage of our HTEMC algorithm is significant. When the number of rules increases to 5000, the average memory usage of HTEMC algorithm reduces by 45% in comparison with PTIAL algorithm.

### Update performance comparison

This part focuses on the cost of the algorithm update. Algorithm updates include adding new rules, deleting or modifying existing rules. We conduct a experiment with 100 rules, and compare the update time on the same rule of the two algorithms. The comparison result is shown in Table 6.

**Table 6:** Update performance comparison (usec / rule).

	PTIAL	HTEMC
**Time of adding rules**	1230	210
**Time of deleting rules**	1240	190
**Time of modifying rules**	1200	105

From Table 6 we can see that the time that our HTEMC algorithm costs when the rule updates is less than PTIAL algorithm. In the process of algorithm updates, HTEMC algorithm is able to quickly find the locations which need to be modified without traversing all the nodes. Thus our HTEMC algorithm is superior to PTIAL algorithm in terms of update performance.

## Performance evaluation in real environment

In this section, we present the experiments to compare the performances of our algorithm with the famous algorithm HD-Cuts[31] and GroupCuts[18] in real environment. In the experiments, the metrics of algorithm performance include time performance which is evaluated as memory access, and the identification precision which is evaluated as the accuracy of the algorithms.

### Experimental environment

**Fig 6:**
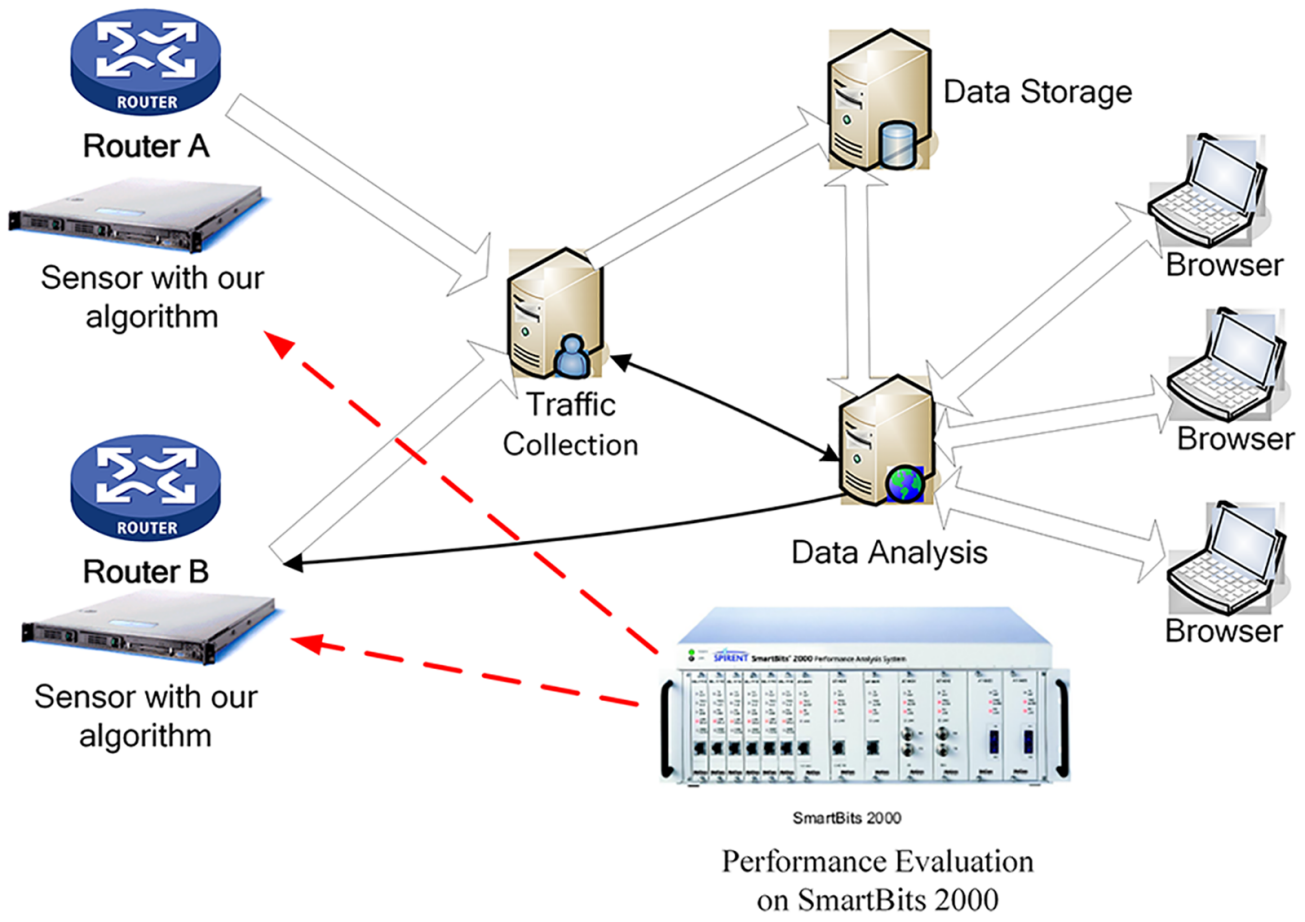
Performance evaluation in real environment.

In order to fully verify the practical performance of the packet classification algorithm, the algorithm and the rule sets should be written on the network traffic monitoring system to test the effectiveness of the algorithms according to the actual network traffic monitoring results. Fig 6 shows the deployment of the network traffic monitoring system at the export link in the campus network. The system is divided into the traffic monitoring sensors, the traffic data collector, the data storage center, the data analysis center and the remote browser. The traffic monitor sensors are deployed in the vicinity of the routers, the network servers and other network equipments. The sensors are responsible for packets mirroring and identifying the packets as the traffic of the application layer. The experimental data of the real network traffic in campus network is acquired by packet classification algorithms in the sensors. In this paper, we use SmartBits 2000 network test platform to test the performance of the algorithms, and further to improve our algorithm in order to enhance the efficiency of the algorithm in practical application.

In the following part, we use two group experiments to test and analyze the performance of the algorithms.

### The evaluation on speed and accuracy

**Fig 7:**
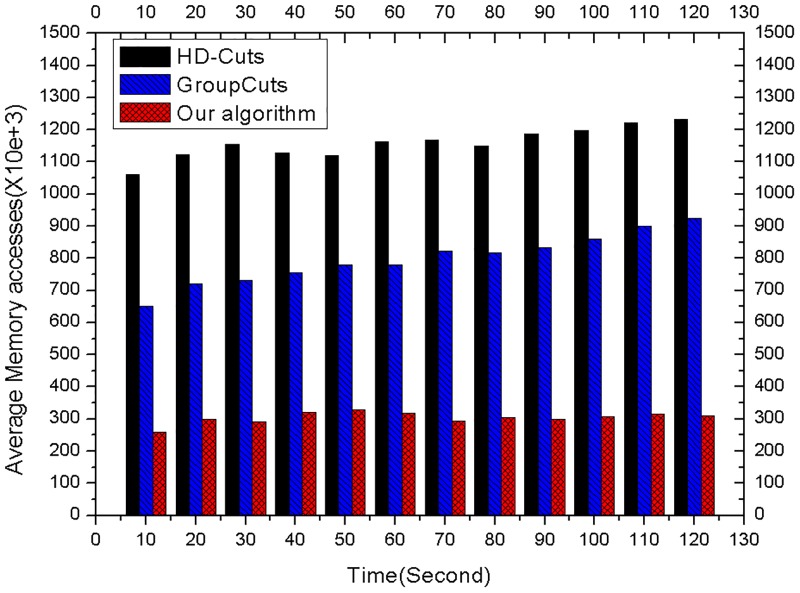
The memory access comparison.

**Fig 8:**
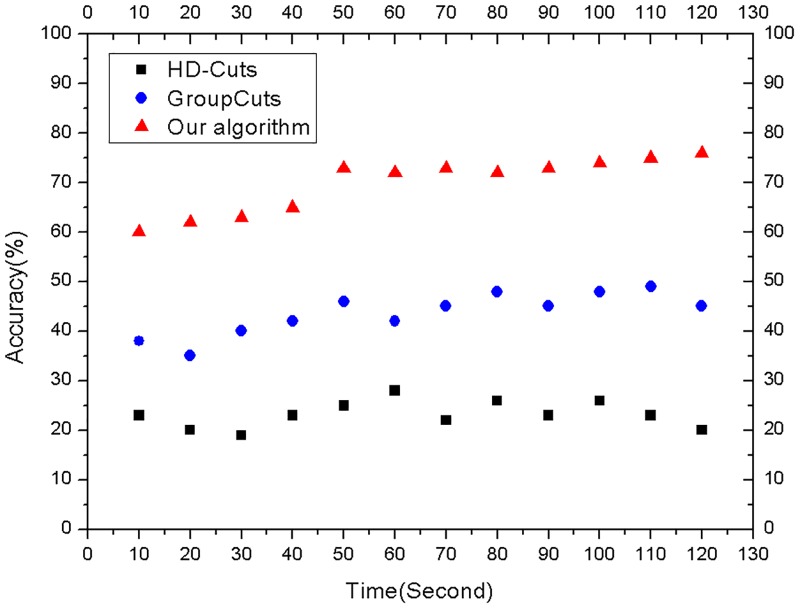
The accuracy comparison.

There are two sets of experiments to respectively test the speed and accuracy performance. The first set of experiments is utilized to evaluate the speed of the three algorithms with the same experimental configuration. As shown in Fig 7, compared with the algorithms HD-Cuts[28] and GroupCuts[18], the average memory accesses of our algorithm separately drop by 73.76% and 61.85%. This result demonstrates that our algorithm has a fast speed to identify the traffic flows. The second set of experiments is to compare the accuracy of the three algorithms. As shown in Fig 8, compared with the accuracy (23.17%) of HD-Cuts algorithm[28] and the accuracy (43.58%) of GroupCuts algorithm[18], our algorithm has a higher accuracy (69.83%). This result demonstrates that our algorithm is more suitable for actual deployment.

## Conclusions

Packet classification algorithms need to deal with a growing size of rule sets with the increasing demand for network bandwidth, nevertheless the existing processing speed cannot meet the development of computer networks. Studies supporting efficient packet classification algorithms for large-scale rule sets are of great significance.

This paper proposed a hierarchical trie algorithm for packet classification based on expectation-maximization clustering. Firstly, we use the formalization method to deal with the packet classification problem. Specifically, we map the rules and data packets into a two-dimensional space. Secondly, we use Expectation-Maximization algorithm to cluster the rules based on their aggregate characteristics, and thereby diversified clusters are formed. Thirdly, we proposes a hierarchical trie based on the results of expectation-maximization clustering. Finally, we respectively conduct simulation experiments and real-environment experiments to compare the performances of classification time and used memory with typical algorithms, and analyze the results of the experiments. By employing the formalization method and expectation-maximization algorithm, our HTEMC algorithm not only adopts trie path compression to eliminate backtracking, but also overcomes the difficulty of update performance, which greatly improve the performance of our algorithm. The experimental results show that our HTEMC algorithm has high-speed packet classification performance, low storage requirement, and is easy to implement and deploy compared with other algorithms.

Although the proposed algorithm has many advantages, such as high searching speed, low storage space and high update speed, it also has some disadvantages. First, the process of constructing a trie is relatively complex so that it needs a certain preprocessing time. The system start time is a little longer, but once the system starts it will run faster. Thus, the proposed algorithm is suitable for the large-scale high-speed network system, and is not suitable for low speed flexible network system. Second, the performance of the proposed algorithm applied in the scenario of huge rule set need to be further tested. For example, when the number of rules is more than 500,000, the performance of the algorithm is unbeknown.

## Supporting information

S1 Table Experiment results of searching speed performances under small sizes of rule sets. (XLSX)

S2 Table Experiment results of searching speed performances under large sizes of rule sets. (XLSX)

S3 Table Experiment results of memory performances under small sizes of rule sets. (XLSX)

S4 Table Experiment results of memory performances under large sizes of rule sets. (XLSX)

S5 Table Experiment results of the memory access comparison among three algorithms. (XLSX)

S6 Table Experiment results of the accuracy comparison among three algorithms. (XLSX)
